# Gender Differences in Trajectories of Depressive Symptoms Among Talkspace Clients: Naturalistic Observational Study

**DOI:** 10.2196/75290

**Published:** 2025-12-03

**Authors:** Meghan Romanelli, Julien Rouvere, Isaac A Sanders, Aarthi Padmanabhan, Emily Peake, Thomas D Hull, Tim Althoff

**Affiliations:** 1 School of Social Work, University of Washington, 4101 15th Avenue NE, Seattle, WA, 98105, United States, 1 206-685-6948; 2 Department of Psychiatry and Behavioral Medicine, University of Washington School of Medicine, Seattle, WA, United States; 3 School of Social Work University of Washington, Seattle, WA, United States; 4 Talkspace, New York, NY, United States; 5 Allen School of Computer Science & Engineering, University of Washington, Seattle, WA, United States

**Keywords:** gender identity, telemedicine, digital mental health interventions, depression, anxiety

## Abstract

**Background:**

Gender minority populations experience an increased risk of depression and report significant barriers to accessing mental health services. While digital mental health (DMH) technologies may address barriers, it remains unclear how gender minority clients engage with DMH services and if DMH improves their clinical outcomes.

**Objective:**

This naturalistic study explored gender differences in 15-week clinical outcomes of clients receiving technology-mediated psychotherapy from a large DMH provider.

**Methods:**

This study used observational data of clients who signed up for Talkspace (Talkspace, Inc) between February 2017 and July 2021. The analytic sample included Talkspace clients (N=20,156) with a baseline 8-item Patient Health Questionnaire (PHQ-8) score ≥10. Participants completed at least 2 PHQ-8 assessments over 15 weeks of treatment. Multilevel linear models tested gender differences in depressive symptom trajectories over the course of treatment (model 1) while also controlling for baseline PHQ-8 scores (model 2) and treatment engagement indicators (model 3). Sensitivity analyses reestimated model 2 among clients who submitted a PHQ-8 survey during the week 15 assessment period and among those who discontinued treatment beforehand. Reasons for service cancellation were also described for the latter group. Gender differences in secondary clinical outcomes were examined via chi-square and Fisher exact tests.

**Results:**

In all models, there were significant week-by-gender interactions. When controlling for baseline PHQ-8 scores, rates of symptom change were significantly slower for gender-diverse participants (*b*=0.60; *P*<.001), nonbinary participants (*b*=0.81; *P*<.001), and transgender women (*b*=0.87; *P*=.007), but not for women (*P*=.98) or transgender men (*P*=.38) compared to men. By week 15, adjusted PHQ-8 scores declined 8.7 points for both men and women, versus 4.4‐7.4 points for gender minority clients. Sensitivity analyses indicated attenuated symptom improvement among week-15 completers, with transgender women showing the slowest changes (*b*=0.76; *P*=.02). Among earlier dropouts, weekly symptom reductions were steep overall (eg, week 3: *b*=−4.06, *P*<.001; week 6: *b*=−2.31, *P*<.001) while certain gender minority subgroups worsened (eg, adjusted scores for transgender women increased from 15.41 at baseline to 16.08 at final week 3 PHQ-8 survey submissions). Cancellation data (3450/20,156, 17.12%) confirmed discontinuation reasons related to both symptom improvement (928/3691 reasons, 25.14%) and potential barriers to treatment engagement (eg, cost: 1431/3691, 38.77%; poor service fit or poor perceived effectiveness: 677/3691, 18.34%). Gender differences were observed in rates of treatment response (weeks 3‐12; all *P*≤.02), symptom remission (weeks 3, 6, 9, and 15; all *P*≤.047), and clinically significant symptom reduction (all time points, all *P*≤.03). Symptom deterioration did not differ by gender (all *P*>.05).

**Conclusions:**

While clinical outcomes generally improved over time among clients engaged in technology-mediated psychotherapy, some gender minority populations experienced slower improvements. Future research may explore strategies to adapt DMH interventions to better meet the needs of diverse gender identities.

## Introduction

Gender minority populations, including transgender, nonbinary, and gender-diverse individuals, experience increased risk of depression relative to cisgender counterparts due to chronic exposure to structural and interpersonal stressors [1,2]. As the leading cause of disability worldwide [3], depression is a serious public health concern. Recent estimates show that 332 million people are diagnosed with depressive disorder globally, resulting in extensive disease burden (ie, economic costs, social costs, morbidity, and mortality) [4-6]. While depression is one of the most common mental health problems, it is also one of the most treatable [7]. However, gender minority populations remain disproportionately impacted. Research indicates that gender minority individuals experience depression at higher rates than cisgender peers [8-11]. For example, in the United States, self-reported lifetime prevalence of depression among transgender populations is close to 30%, which far exceeds cisgender rates (18%) [12]. Electronic health records confirm this disparity with higher rates of depressive disorder diagnoses observed among gender minorities relative to cisgender counterparts (cisgender men: 16.8%; cisgender women: 23.7%; transgender men: 29.5%; transgender women: 27%; and nonbinary and gender diverse: 32.2%) [13].

Despite the increased need for mental health care, gender minority populations report significant barriers to accessing services [14,15]. For example, the availability of affirmative services is limited. Affirmative services are responsive to the unique stressors and needs of gender minority clients, providing care that is associated with greater treatment satisfaction and treatment effectiveness [16]. These services, however, are not only few in number but are also primarily clustered in urban enclaves, further restricting access to care for a geographically dispersed population [17,18]. Access barriers can result in forgone care, restrict treatment engagement, and lead to significant unmet mental health needs [19]. Indeed, gender minority populations face large treatment gaps. For example, research indicates that transgender people are 2.4 times more likely than their cisgender counterparts to report unmet mental health needs, including untreated depression [20].

Digital mental health (DMH) technologies are promising to address the barriers of traditional service delivery models that restrict marginalized populations’ access to mental health resources [21-23]. DMH may offer flexible and remote treatment options [21] that allow clients to engage with licensed therapists through text, audio, or video messages [24]. Digital resources are an essential source of information and connection among gender minority populations, especially those who report isolation and discrimination [25]. Gender minority care seekers increasingly prefer to seek online support and professional health services [26-28], citing benefits of self-directed care, confidentiality, anonymity, and safety [23,27,29,30]. Therefore, DMH options present an acceptable mode of treatment delivery with service qualities that align with community needs and preferences. Despite potential, it remains unclear how gender minority consumers engage with DMH services and if DMH effectively improves their clinical outcomes [23,29,31].

This naturalistic study explored the gender differences in longitudinal treatment engagement and 15-week clinical outcomes among clients receiving technology-mediated psychotherapy from a large DMH provider. First, this study characterized clients and their engagement in care, including treatment retention and weekly message and word counts. Next, this study examined gender differences in trajectories of depressive symptoms over a 15-week period. Gender minority clients often face unmet mental health needs, but DMH may lead to better outcomes by reducing barriers and providing more affirming options. Given these competing possibilities, analyses were approached as exploratory and focused on potential heterogeneity in depressive symptom trajectories by gender. Ultimately, the research endeavored to provide insights that can inform the development and implementation of effective DMH services that address critical mental health and access disparities experienced by gender minority populations. This study was conducted following the STROBE (Strengthening the Reporting of Observational Studies in Epidemiology) reporting guidelines (Checklist 1) [32].

## Methods

### Design and Procedures

This study used longitudinal observational data of clients who signed up for Talkspace (Talkspace, Inc) between February 2017 and July 2021. Data included in this study were routinely collected as part of the service. Talkspace [33] is a telemedicine platform of over 5000 licensed therapists across the United States. Clients access the platform through the internet and complete a brief intake with a consulting clinician to identify their presenting problems and treatment history. The intake clinician also helps the client select their service plan (eg, asynchronous messaging only; asynchronous messaging plus monthly or weekly 30-minute synchronous video sessions) and records information about therapist preferences (eg, gender and language preferences). Using this information, a matching algorithm selects 3 clinicians whose expertise and availability best align with the client’s preferences and treatment needs. Clients review clinician information and self-select a clinician for continued treatment. This process ensures agency and self-direction, principles that align with preferred service qualities identified by gender minority consumers [23,27,29,30].

### Participants

All participants of the current study were Talkspace clients engaged in technology-mediated individual psychotherapy. Each participant completed at least two 8-item Patient Health Questionnaire (PHQ-8) assessments, including a baseline survey after clinician selection and, minimally, 1 additional PHQ-8 survey at a subsequent time point. Inclusion criteria included a baseline PHQ-8 score of ≥10, that clients were English-speaking, US residents, had internet or smartphone access, and provided information on gender. The final analytic sample included data from 20,156 participants. Participants worked with 3804 different therapists contracted or employed by Talkspace, all of whom were Master’s level or higher, licensed clinicians.

### Intervention

Therapists and clients exchanged asynchronous text, audio, or video-based messages depending on client preference, and if available through the service plan, attended weekly or monthly 30-minute live video sessions. Unlimited and 24/7 asynchronous messaging was available for all clients. Therapists reviewed client messages and responded at least once per day during standard workdays and hours. All messaging occurred over a secure, Health Insurance Portability and Accountability Act (HIPAA)–compliant platform that could be accessed on mobile and desktop devices.

### Measures

#### Participant Characteristics: Gender

Demographic characteristics were collected at baseline. All clients included in the analytic sample reported their gender at sign-up by answering a single question, “What gender do you identify with?” (male, female, transgender male, transgender female, nonbinary, gender queer, gender variant, or other). Because gender was assessed with a single item, it cannot be definitively determined that clients who selected “male” or “female” identify as cisgender. Therefore, the term “cisgender” was avoided in reporting study results as it is often incorrectly applied as an assumed default label for individuals reporting their gender as male or female [34,35]. In this study, clients who indicated their gender as gender queer (142/20,156, 0.70%), gender variant (25/20,156, 0.12%), or “other” (44/20,156, 0.22%) are grouped as “gender diverse.” This identification reflects terminology suggested by the National Academies of Engineering, Sciences, and Medicine that inclusively encompasses a spectrum of identities that do not align with traditional gender norms. Henceforth, we will also use terminology to reflect gender (men, women, transgender men, and transgender women) rather than sex (male, female, transgender male, and transgender female) [35].

#### Additional Descriptive Characteristics

Additional sociodemographic characteristics were optionally answered by clients to describe participants’ age in years and race or ethnicity (African American, Asian, Caucasian, Hispanic, Native American, other race, declined to respond, and no response).

#### Engagement Indicators: Messaging

The total number of messages and the total number of words that clients sent to their therapist were calculated weekly throughout the duration of treatment on the platform. Client *message count* and *word count* are both automatically computed by the platform and stored as metadata. In this study, each week that a client exchanged any message with their therapist was considered a week of *active messaging*. The total number of active messaging weeks was calculated for the 15-week study period and across a client’s time on the platform.

#### Therapist Change

Platform clients can switch therapists at any time. In this study, a therapist change was identified by examining client ID-therapist ID pairings across treatment weeks through the study period. Any participant who was paired with multiple therapists was coded as changing therapists (0=no; 1=yes).

#### Depressive Symptoms

Depressive symptoms were measured with the PHQ-8 [36]. This brief, self-report survey assesses the severity of depressive symptoms, incorporating criteria from the *Diagnostic and Statistical Manual, Fourth Edition*. The PHQ-8 excludes an item evaluating suicidality. Items are rated on a 4-point scale (0=not at all to 3=nearly every day) and summed to calculate a total score, with higher scores indicating greater depressive symptom severity. A total score ≥10 indicates clinically significant depression.

Clients were prompted to complete the PHQ-8 via the platform app at treatment initiation and subsequently every 3 weeks through service termination. A buffer was permitted such that clients could submit the PHQ-8 1 week before or after the target assessment week. Time points of this study included target assessment week 0 (full baseline), week 3, week 6, week 9, week 12, and week 15. While most participants recorded their first PHQ-8 prior to treatment at week 0 (17,622/20,156; 87.4%), a small proportion submitted their first survey at target assessment week 3 (2534/20,156; 12.6%). All participants within this latter group also returned at least 1 additional requisite PHQ-8 survey at a subsequent assessment time point. Survey completion was not required for service continuation.

#### Secondary Clinical Outcomes

Four additional measures of depressive symptom change were examined, including treatment response, symptom remission, clinically significant symptom reduction, and symptom deterioration. Prior studies of Talkspace data have assessed these secondary clinical outcomes [37], which align with common definitions of meaningful change in depressive symptoms.

#### Treatment Response

Differences between PHQ-8 scores at baseline and individual follow-up assessment time points were calculated. Clients were identified as responsive to treatment if this difference represented a 50% or larger reduction in symptoms (0=no; 1=yes). This is a widely accepted indicator of meaningful symptom improvement, supported by PHQ-8 validation studies [36,38] and methodological comparison research [39, 40].

#### Symptom Remission

Symptom remission was identified if the PHQ-8 score, measured at a follow-up assessment time point, was less than 5 (0=no; 1=yes). This threshold is commonly used to indicate the absence of depressive symptoms and aligns with established definitions of remission from depression in clinical and research contexts [36,39].

#### Clinically Significant Symptom Reduction

Clients were identified as having a clinically significant reduction in depressive symptoms if, at a follow-up time point, their PHQ-8 score was below the clinical threshold (<10) *and* their score improved by at least 5 points from the client’s baseline (0=no; 1=yes). This compound metric indicates both a reliable change in depressive symptoms and a shift below the threshold for clinically significant depression [38-40].

#### Symptom Deterioration

Clients’ depressive symptoms were identified as deteriorating if their PHQ-8 scores increased by 5 or more points from their baseline at a follow-up assessment time point (0=no; 1=yes). This increase marks a reliable and significant change consistent with an exacerbation of depressive symptoms [39,40].

### Analytic Plan

All analyses were completed in Stata (version 19.5; StataCorp). Distributions of participant sociodemographic characteristics, engagement indicators, and depressive symptoms were estimated across the sample and stratified across gender categories. While the primary analyses for the multilevel linear models analyzed 15-week clinical outcomes, a description of engagement indicators for the sample’s full course and adjusted 15-week treatment data are both reported. As reported elsewhere [41], few Talkspace clients with clinically significant PHQ-8 scores at baseline remain in treatment past week 16. These analyses suggested that some clients discontinued after early treatment gains (eg, average PHQ-8 scores fell below the clinical cut-off by week 6), while others’ disengagement was associated with factors unrelated to symptom remission (eg, therapist’s years of experience; first-time therapy client).

Multilevel linear models with maximum likelihood estimation and robust SEs were used to examine changes in depressive symptoms over 15 weeks of treatment. This approach was chosen to account for the nested data structure of clients’ repeated PHQ-8 measures. Because clients could switch therapists throughout their treatment, and therapists could treat multiple clients on the platform, a cross-classified model was initially considered to accurately reflect the nature of the data structure by partitioning the unique variance attributable to both clients and therapists [42]. However, an examination of the unconditional models indicated that therapist-level effects did not significantly explain additional variance in PHQ-8 scores relative to client-level effects, and models had poorer fit statistics, suggesting unnecessary complexity and potential overfitting. Considering fit, parsimony, and interpretability, the final models included only client-level random intercepts and random slopes for time.

The first model tested the interaction effects of gender and treatment week on depressive symptoms. This analysis examined differences in PHQ-8 scores over the course of treatment between men, women, transgender men, transgender women, and nonbinary and gender-diverse clients. The second model also controlled for baseline PHQ-8 scores. The third model further incorporated engagement indicators, including therapist change as a predictor and the interaction effects of assessment time point and number of weeks of active messaging. This interaction analysis aimed to clarify whether engagement, through consistent client-therapist correspondence throughout treatment, and time-varying treatment effects had differential impacts on depressive symptoms. Week number was a continuous variable (full baseline or week 0=0; week 3=1; week 6=2; week 9=3; week 12=4; week 15=5). In all regression models, clients who identified as men served as the reference group, as they have historically shown the lowest levels of depressive symptoms among the gender subgroups included in this study. However, estimated marginal means were calculated and plotted for each assessment time point and gender subgroup to clarify and depict depressive symptom trajectories for every gender subgroup.

Prevalence of the secondary clinical outcomes (ie, treatment response, symptom remission, clinically significant symptom reduction, and symptom deterioration) was calculated for each gender subgroup at each PHQ-8 assessment time point. Differences in secondary outcomes between groups were examined via the chi-square and Fisher exact tests.

### Ethical Considerations

All data were collected for the service provider’s quality assurance and program management. As part of the platform’s regular service use agreement, both clients and therapists provided consent for data use in deidentified, aggregate form for research purposes. Therefore, participants were not compensated for their involvement in the study. All materials and procedures for this study were approved by the University of Washington institutional review board, including future secondary analysis of data without direct identifiers (STUDY00010958).

## Results

### Descriptive Statistics

The mean age of the sample was 30.52 (SD 8.65) years. Most participants identified as women (15,334/20,156, 76.08%), followed by men (4411/20,156, 21.88%), gender-diverse participants (211/20,156, 1.05%), nonbinary participants (104/20,156, 0.52%), transgender men (67/20,156, 0.33%), and transgender women (29/20,156, 0.14%). Across all participants, clinically significant levels of depressive symptoms were identified at baseline, with scores averaging 15.16 (SD 3.79). Throughout the complete study period, transgender, nonbinary, and gender-diverse participants averaged higher depression severity (mean 12.97‐14.45, SD 5.07-5.20) compared to women and men (mean 11.86, SD 5.54 and mean 11.88, SD 5.62, respectively). Transgender women had the most weeks of active messaging in total (mean 21.41, SD 20.90) and during the study period (mean 11.48, SD 4.98). Transgender men had the fewest weeks of active messaging in total (mean 15.58, SD 20.65) and during the study period (mean 9.57, SD 4.82; Table 1).

**Table 1.** Baseline demographic, engagement, and depressive symptom characteristics of Talkspace clients (2017‐2021; N=20,156).

	Total sample	Transgender men (n=67)	Transgender women (n=29)	Nonbinary (n=104)	Gender diverse (n=211)	Women (n=15,334)	Men (n=4411)
Race or ethnicity, n (%)							
African American	360 (1.79)	0 (0)	0 (0)	1 (0.96)	2 (0.95)	307 (2.00)	50 (1.13)
Asian	208 (1.03)	0 (0)	0 (0)	1 (0.96)	1 (0.47)	165 (1.08)	41 (0.93)
Caucasian	1778 (8.82)	10 (14.93)	4 (13.79)	3 (2.88)	15 (7.11)	1334 (8.70)	412 (9.34)
Hispanic	217 (1.08)	0 (0)	0 (0)	0 (0)	3 (1.42)	158 (1.03)	56 (1.27)
Native American	10 (0.05)	0 (0)	0 (0)	0 (0)	0 (0)	9 (0.06)	1 (0.02)
Biracial	26 (0.13)	0 (0)	0 (0)	0 (0)	3 (1.42)	20 (0.13)	3 (0.07)
Other	228 (1.13)	0 (0)	1 (3.45)	2 (1.92)	2 (0.95)	171 (1.12)	52 (1.18)
Declined	41 (0.20)	0 (0)	0 (0)	1 (0.96)	1 (0.47)	34 (0.22)	5 (0.11)
No response	17,228 (85.77)	57 (85.07)	24 (82.76)	96 (92.31)	184 (87.20)	13,136 (85.67)	3791 (85.94)
Age (y; n=15,716), mean (SD)	30.52 (8.65)	24.27 (8.08)	27.88 (7.09)	25.16 (7.55)	26.95 (7.69)	30.35 (8.58)	31.57 (8.76)
Engagement							
Therapist Change, n (%)	1683 (8.35)	0 (0)	1 (3.45)	7 (6.73)	10 (4.74)	1269 (8.28)	396 (8.98)
Weekly message count, mean (SD)	13.34 (23.94)	10.44 (13.96)	13.10 (16.63)	14.99 (21.66)	13.75 (18.56)	13.27 (24.94)	13.58 (20.56)
Weekly word count, mean (SD)	1002.64 (1434.11)	792.03 (1151.00)	1060.54 (1279.96)	928.05 (1311.81)	1127.76 (1550.77)	998.74 (1379.10)	1014.84 (1614.01)
Weeks of active messaging (total)^a^, mean (SD)	16.62 (20.70)	15.58 (20.65)	21.41 (20.90)	16.91 (15.17)	19.86 (26.14)	16.68 (20.83)	16.23 (20.04)
Weeks of active messaging (study period)^b^, mean (SD)	10.29 (4.78)	9.57 (4.82)	11.48 (4.98)	10.81 (4.76)	10.42 (4.81)	10.29 (4.78)	10.26 (4.78)
PHQ-8^c^, mean (SD)							
Baseline score	15.16 (3.79)	16.69 (4.05)	15.90 (3.43)	15.26 (3.76)	15.45 (3.78)	15.15 (3.77)	15.14 (3.82)
Average score across study period	11.90 (5.55)	14.45 (5.20)	13.1 (5.45)	12.97 (5.07)	13.2 (5.15)	11.86 (5.54)	11.88 (5.62)

a Weeks of active messaging (total): total number of weeks that a client exchanged messages with their therapist during the entire period of time they were in treatment on the Talkspace platform.

b Weeks of active messaging (study period): total number of weeks that a client exchanged messages with their therapist, not exceeding week 15. If weeks of active messaging (total)>week 15 then weeks of active messaging (study period)=15.

c PHQ-8: 8-item Patient Health Questionnaire.

### Depressive Symptom Trajectories by Gender and Treatment Engagement

**Figure 1.**
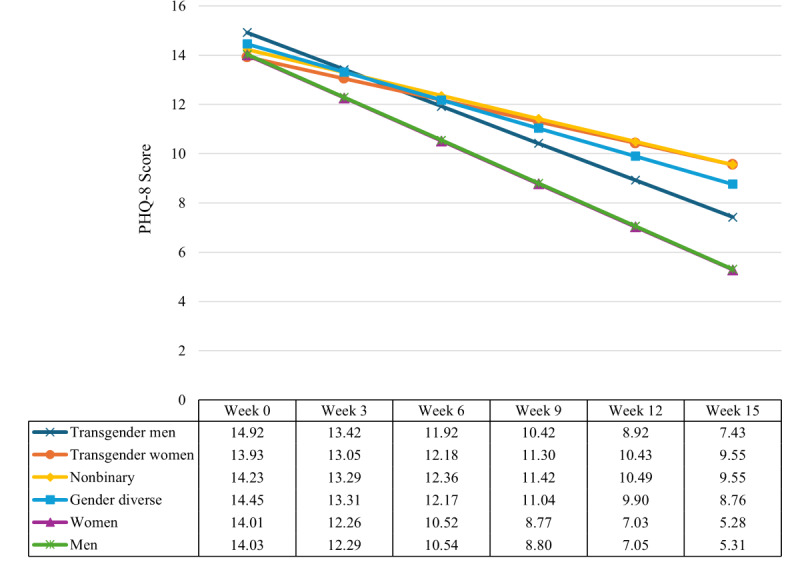
Adjusted 15-week depressive symptom trajectories by gender estimated among Talkspace clients (2017‐2021). PHQ-8: 8-item Patient Health Questionnaire.

Table 2 displays model unstandardized (*b*) and standardized regression (β) coefficients and fit indices for models 1 (base model), 2 (primary model; controlling for intake PHQ-8), and 3 (controlling for therapist change, weeks of active messaging, and week-by-weeks of active messaging). In all models, there were significant week-by-gender interactions, indicating that changes in depressive symptoms over the course of treatment varied by gender. In model 2, the rate of change in PHQ-8 scores by treatment week for women (*b*=–.0009; *P*=.98) and transgender men (*b*=.25; *P*=.38) were not significantly different from that of men. Compared to men, rates of change were significantly different for gender-diverse participants (*b*=0.60; *P*<.001). The week-by-gender interactions for nonbinary participants (*b*=0.81; *P*<.001) and transgender women (*b*=0.87; *P*=.007) were significant, with both improving at the slowest rates among the gender subgroups. In model 3, therapist change, weeks of active messaging, and the interaction of week-by-weeks of active messaging were statistically significant (*P*<.001), but these additions only had a small impact on model fit. Inclusion of these covariates resulted in lower estimates for the week-by-gender interactions (ie, attenuated change in PHQ-8 scores over time compared to model 2). Figure 1 visualizes the week-by-gender interaction for model 2.

**Table 2.** Multilevel linear models of depressive symptom trajectories among Talkspace clients (2017‐2021).

	Model 1^a^				Model 2^b^				Model 3^c^			
	*b^d^*	SE	β^e^	*P* value	*b*	SE	β	*P* value	*b*	SE	β	*P* value
Intercept	14.07	0.06	—^f^	—	2.34	0.08	—	—	3.34	0.09	—	—
Week number	−1.61	0.03	−.42	<.001	−1.74	0.04	−.45	<.001	−3.34	0.07	−.49	<.001
Gender^g^												
Transgender men	1.98	0.50	.43	<.001	0.89	0.22	.22	<.001	0.63	0.21	.21	.002
Transgender women	0.30	0.73	.29	.68	-0.10	0.36	.21	.78	−0.23	0.38	.18	.54
Nonbinary	0.40	0.37	.25	.28	0.20	0.24	.25	.42	0.21	0.23	.23	.35
Gender diverse	0.63	0.30	.26	.03	0.42	0.16	.24	.008	0.36	0.15	.21	.02
Women	−0.04	0.07	−.002	.55	−0.02	0.04	−.004	.63	−0.03	0.04	−.008	.44
Week number x gender^g^												
Week number x transgender men	0.30	0.27	.08	.28	0.25	0.28	.06	.38	0.36	0.27	.09	.18
Week number x transgender women	0.88	0.29	.23	.003	0.87	0.32	.23	.007	0.82	0.32	.21	.01
Week number x nonbinary	0.67	0.20	.18	.001	0.81	0.20	.21	<.001	0.72	0.21	.19	<.001
Week number x gender diverse	0.57	0.14	.15	<.001	0.60	0.15	.16	<.001	0.57	0.15	.15	<.001
Week number x women	0.02	0.04	.01	.58	−0.0009	0.04	−.0002	.98	-0.01	0.04	−.002	.84
Baseline PHQ-8^h^ score	—	—	—	—	0.77	0.005	.52	<.001	0.77	0.005	.520	<.001
Therapist change, yes^i^	—	—	—	—	—	—	—	—	−1.58	0.08	-.28	<.001
Weeks of active messaging (study period)	—	—	—	—	—	—	—	—	−0.07	0.003	.10	<.001
Week number x weeks of active messaging (study period)	—	—	—	—	—	—	—	—	0.13	0.004	.16	≤.001

a Model fit statistics for model 1: Akaike information criterion (AIC) 361,597.8; Bayesian information criterion (BIC) 361,733.0; −2LL=−180,783.9, df=15; conditional *R*2=0.55; marginal *R*2=0.16.

b Model fit statistics for model 2: AIC 345,509.1; BIC 344,004.2; −2LL=−172,738.6, df=16; conditional *R*2=0.71; marginal *R*2=0.36.

c Model fit statistics for model 3: AIC 345,653.3; BIC 344,175.4; −2LL=−171,983.1, df=19; conditional *R2*=0.71; marginal *R2*=0.35.

d *b* represents the raw or unstandardized regression coefficient.

e β represents the standardized regression coefficient (β). Standardized regression coefficients were derived from unstandardized estimates following guidance from Hox [43]. In multilevel models, these values are approximate effect sizes only and should be interpreted with caution as standardization does not uniquely partition variance across levels.

f Not applicable.

g Reference group: men.

h PHQ-8: 8-item Patient Health Questionnaire.

i Reference group: no.

### Secondary Clinical Outcomes

#### Overview

All secondary clinical outcomes across the study period are reported in Table 3. Results indicated differential rates of treatment response, symptom remission, and clinically significant symptom change by gender.

**Table 3.** Secondary clinical outcomes across 15 weeks by gender among Talkspace clients (2017‐2021).^a^

	Full sample	Transgender women	Transgender men	Nonbinary	Gender diverse	Women	Men	*P* value^b^
Week 3								
Available, n	15,489^c^	19^c^	54^c^	78^c^	161^c^	11,807^c^	3370^c^	—^d^
Treatment response^e^, n (%)	4694 (30.31)	4 (21.05)	7 (12.96)	15 (19.23)	32 (19.88)	3624 (30.69)	1012 (30.03)	<.001
Symptom remission^f^, n (%)	1984 (12.81)	1 (5.26)	2 (3.70)	9 (11.54)	10 (6.21)	1538 (13.03)	424 (12.58)	.03
Clinically significant change^g^, n (%)	5580 (36.03)	5 (26.32)	8 (14.81)	17 (21.79)	42 (26.09)	4329 (36.66)	1179 (34.99)	<.001
Symptom deterioration^h^, n (%)	608 (3.93)	0 (0)	2 (3.70)	6 (7.69)	6 (3.73)	448 (3.79)	146 (4.33)	.35
Week 6								
Available, n	10,240	20	39	62	114	7829	2176	—
Treatment response^e^, n (%)	3518 (34.36)	8 (40.00)	10 (25.64)	12 (19.35)	22 (19.30)	2700 (34.49)	766 (35.20)	.001
Symptom remission^f^, n (%)	1643 (16.04)	4 (20.00)	2 (5.13)	5 (8.06)	9 (7.89)	1259 (16.08)	364 (16.73)	.02
Clinically significant change^g^, n (%)	4092 (39.96)	8 (40.00)	10 (25.64)	12 (19.35)	26 (22.81)	3169 (40.48)	867 (39.84)	<.001
Symptom deterioration^h^, n (%)	402 (3.93)	0 (0)	1 (2.56)	6 (9.68)	6 (5.26)	294 (3.76)	95 (4.37)	.15
Week 9								
Available, n	6161	16	17	39	67	4719	1303	—
Treatment response^e^, n (%)	2345 (38.06)	4 (25.00)	3 (17.65)	8 (20.51)	18 (26.87)	1819 (38.55)	493 (37.84)	.02
Symptom remission^f^, n (%)	1181 (19.17)	1 (6.25)	1 (5.88)	3 (7.69)	7 (10.45)	904 (19.16)	265 (20.34)	.05
Clinically significant change^g^, n (%)	2661 (43.19)	4 (25.00)	4 (23.53)	9 (23.08)	22 (32.84)	2070 (43.87)	552 (42.36)	.008
Symptom deterioration^h^, n (%)	246 (3.99)	0 (0)	0 (0)	3 (7.69)	3 (4.48)	188 (3.98)	52 (3.99)	.77
Week 12								
Available, n	4310	10	15	28	51	3269	937	—
Treatment response^e^, n (%)	1782 (41.35)	2 (20.00)	5 (33.33)	11 (39.29)	9 (17.65)	1363 (41.69)	392 (41.84)	.009
Symptom remission^f^, n (%)	937 (21.74)	2 (20.00)	1 (6.67)	5 (17.86)	4 (7.84)	717 (21.93)	208 (22.20)	.12
Clinically significant change^g^, n (%)	1987 (46.10)	2 (20.00)	6 (40.00)	11 (39.29)	11 (21.57)	1511 (46.22)	446 (47.60)	.004
Symptom deterioration^h^, n (%)	185 (4.29)	1 (10.00)	2 (13.33)	2 (7.14)	4 (7.84)	137 (4.19)	39 (4.16)	.13
Week 15								
Available, n	2975	13	10	20	37	2243	652	—
Treatment response ^e^, n (%)	1224 (41.14)	3 (23.08)	3 (30.00)	8 (40.00)	9 (24.32)	921 (41.06)	280 (42.94)	.19
Symptom remission^f^, n (%)	665 (22.35)	1 (7.69)	1 (10.00)	2 (10.57)	4 (10.81)	487 (21.71)	170 (26.07)	.03
Clinically significant change^g^, n (%)	1368 (45.98)	5 (38.46)	2 (20.00)	8 (40.00)	9 (24.32)	1027 (45.79)	317 (48.62)	.03
Symptom deterioration^h^, n (%)	127 (4.27)	1 (7.69)	0 (0)	0 (0)	2 (5.41)	94 (4.19)	30 (4.60)	.79

a Scores calculated using observed 8-item Patient Health Questionnaire (PHQ-8) scores.

b *P* value reported for the chi-square or the Fisher exact when n<5.

c Available data for week 3 calculations excluded cases whose first PHQ-8 survey was returned during the week 3 assessment time point as secondary clinical outcome calculations required baseline data.

d Not applicable.

e Treatment response rate: 50% or larger reduction in symptoms from baseline.

f Symptom remission: PHQ-8 score <5 at assessment time point.

g Clinically significant symptom change: below clinical threshold (PHQ-8 score<10) and improving at least 5 points from baseline.

h Symptom deterioration: PHQ-8 score worsened by ≥5 points from baseline.

#### Treatment Response

Treatment response rates were significantly different by gender from weeks 3 to 12 (all *P*≤.02). At week 3, treatment response rates were highest for women (3624/11,807, 30.69%) and men (1012/3370, 30.03%) and were ≤21.05% for gender minorities. Transgender men had the lowest treatment response rate (7/54, 12.96%) at week 3. Treatment response rates were not significantly different between gender subgroups at week 15 (*P*=.19).

#### Symptom Remission

There were statistically significant differences in symptom remission rates by gender at weeks 3, 6, 9, and 15 (all *P*≤.047). Differences in remission rates at week 3 (1538/11,807, 13.03% and 424/3370, 12.58% for women and men, respectively; 3.70%‐11.54% for gender minorities) became more pronounced by week 15. Remission rates at week 15 for women and men (487/2243, 21.71% and 170/652, 26.07%, respectively) were over 2 times greater than those of gender minority participants (7.69%‐10.81%).

#### Clinically Significant Symptom Reduction

Rates of clinically significant symptom reduction were significantly different by gender at each time point (all *P*≤.03). Clinically significant symptom reduction rates for transgender women, nonbinary, and gender-diverse clients at week 3 ranged from 21.79% (17/78) to 26.32% (5/19). Rates of women and men with clinically significant symptom reduction at week 3 (4329/11,807, 36.66% and 1179/3370, 34.99%, respectively) were over 2 times greater than that of transgender men (8/54, 14.81%). Clinically significant symptom reduction increased for all groups at week 15 (20%‐40% for gender minorities; 1027/2243, 45.79% and 317/652, 48.62% for women and men, respectively).

#### Symptom Deterioration

There were no significant differences in deterioration rates by gender from weeks 3 to 15 (all *P*>.05). Deterioration rates across all weeks ranged from 3.99% (52/1303) to 4.60% (30/652) for men, 3.76% (294/7829) to 4.19% (137/3269) for women, and 0% to 13.33% for gender minorities.

#### Sensitivity Analysis

Clients displayed high rates of treatment dropout throughout the 15-week study period. Of the 20,156 clients, 2925 (14.5%) completed a week-15 PHQ-8 survey, and 17,231 (85.5%) did not. It is possible that those who remained engaged throughout the entire study period provide a more accurate picture of effectiveness. To examine whether attrition may have biased the estimation of depressive symptom trajectories, a series of sensitivity analyses were conducted.

#### Clients Who Completed the 15-Week Study Period

Model 2 was reestimated using only data from the subgroup of clients who submitted a PHQ-8 survey during the week 15 assessment period (Multimedia Appendix 1). Consistent with the main analysis, these clients experienced significant weekly reductions in depressive symptoms (*b*=−1.01; *P*<.001), although rates were slightly attenuated compared to the main analysis (*b*=−1.74; *P*<.001; Table 2). The week-by-gender interaction was only significant for transgender women (*b*=0.76; *P*=.022). Estimated depressive symptom trajectories were visualized, showing that transgender women who remained engaged through the 15-week study period experienced slower symptom reduction than other gender subgroups (Multimedia Appendix 2).

#### Clients Grouped by Final PHQ-8 Assessment Week

To further contextualize why trajectories estimated in the main analysis differed from those estimated among week-15 completers, model 2 was also reestimated separately among clients grouped by final PHQ-8 assessment week (ie, the last week a PHQ-8 score was available for each client, indicating the final survey they completed; Multimedia Appendix 3). Overall, models of the earliest dropout weeks showed steep weekly symptom change (week 3: *b*=−4.06, *P*<.001; week 6: *b*=−2.31, *P*<.001). However, trajectories varied by gender among some final assessment week groups. For example, among those whose final PHQ-8 was submitted during the week 3 assessment period, transgender women’s PHQ-8 scores increased from baseline to week 3 (Multimedia Appendix 4). Other gender minority clients showed slower rates of improvement, including nonbinary clients whose final PHQ-8 was submitted in week 6 (*b*=1.12; *P*=.03), week 9 (*b*=1.30; *P*=.002), and week 12 (*b*=0.71; *P*=.048) and gender-diverse clients whose final PHQ-8 was submitted in week 6 (*b*=0.87; *P*=.013) and week 12 (*b*=0.87, *P*=.006; MultimediaAppendices 3  4).

#### Reasons for Service Cancellation Before Week 15

Reasons for service cancellation were also examined among the 17,231 clients who discontinued treatment before week 15. Among these clients, 3450 (20.02%) provided at least 1 reason for cancellation (Multimedia Appendix 5). Over a third (1431/3691 reasons, 38.77%) cited reasons related to cost, a quarter reported that they felt better or met their goals (928/3691 reasons, 25.14%), and 18% (677/3691 reasons) indicated poor service fit or poor perceived effectiveness. Higher rates of nonbinary clients reported cost reasons, while higher rates of gender-diverse clients canceled due to issues of fit or perceived effectiveness. Early dropouts may reflect both symptom improvement and barriers to treatment engagement.

## Discussion

### Principal Findings

This study examined gender differences in depressive symptom trajectories and engagement patterns among clients of a DMH platform with clinically significant levels of depression. Data were observational, with outcomes assessed in a naturalistic setting as part of the routine delivery of services, including at baseline and every 3 weeks over 15 weeks of the study period. Results suggested that technology-mediated psychotherapy may reduce depressive symptoms, but that treatment effects over time may function differently across genders. Across 15 weeks, heterogeneous depressive symptom trajectories and differential rates of treatment response, symptom remission, and clinically significant symptom change indicated that some gender minority groups, particularly transgender women, nonbinary, and gender-diverse clients, may not benefit from this model of DMH as immediately as men- and women-identified clients. These findings may have implications for service delivery, highlighting the need for tailored approaches. Combining the broad accessibility of DMH with tailored strategies may better address the mental health needs of gender minority clients.

Transgender women, nonbinary, and gender-diverse clients experienced slower symptom improvement over the 15 weeks of treatment compared to men and women, who each showed similar rates of improvement. These patterns were reflected in significant week-by-gender interactions across all models. We also found that men and women showed more consistent improvements in secondary clinical outcomes. Gender minority individuals face unique stressors that impact their mental health, including chronic exposure to interpersonal and structural stigma, discrimination, and victimization across multiple contexts [44]. Research shows that the cumulative effects of these experiences are associated with poor mental health outcomes [45-48], including longitudinal depressive symptom trajectories [49].

Gender-based discrimination can also typify gender minority individuals’ care-seeking experiences, as they often experience multiple forms of discrimination [19], across multiple settings [50], and at higher rates than cisgender individuals [51]. Experiences of health care discrimination can create hesitancies for open communication with providers. These hesitancies can subsequently disrupt therapeutic alliance and engagement [52,53], which are key factors in improving treatment outcomes for gender minority clients [54,55]. In this study, we found that some gender minority clients actively messaged with their therapist for more weeks and, on average, sent more weekly messages and words compared to men and women. Transgender women and gender-diverse clients, for example, had the highest weekly word count and longest durations of active messaging, but at the same time, their symptom improvement did not keep pace with men and women who messaged less and for shorter durations. While it is possible that these clients required longer periods of messaging due to more complex treatment needs, it is also possible that technology-mediated psychotherapy, as deployed, could not fully address these needs. Consistent with this interpretation, sensitivity analyses indicated that transgender women who remained engaged through all 15 weeks experienced slower symptom improvement than other gender subgroups. Their adjusted PHQ-8 scores at week 15 showed less than a 2-point improvement from baseline and remained above the clinically significant threshold. These findings reinforce that longer engagement may not necessarily yield better clinical outcomes for some gender minority clients.

Furthermore, model 3 found that longer periods of messaging may reflect diminishing returns over time. Early gains in symptom improvement plateaued as messaging continued, possibly due to a weaker rapport limiting therapeutic depth. However, randomized clinical trials of message-based care have yet to demonstrate any differences relative to video-based telehealth [56-58]. For those with prior experiences of discrimination, the delivery format may not have fostered the rapport necessary to support open communication. Messaging content, however, remains unknown and could provide important insights into the unique barriers and needs of gender minority clients. In traditional face-to-face therapy, engagement is more robustly assessed by more than just attendance; active participation and openness are critical to therapeutic progress. Recent research on digital therapeutic alliance emphasizes limitations in replicating cornerstone alliance dimensions during digital services delivery, including bond and empathy [59-61]. Future research should evaluate not only the frequency and duration of messaging but also the quality of engagement to better understand mechanisms of the therapeutic process in the digital context, especially for gender minority clients. In addition, leveraging artificial intelligence tools [62] that integrate into DMH platforms may support therapists’ confidence in delivering empathetic and gender-affirming messages.

Recognizing the importance of rapport in the therapeutic process, Talkspace has integrated a feature that allows clients to easily change their provider for any reason to enhance fit. We found that therapist change was associated with decreased PHQ-8 scores, indicating the benefits of this feature. However, fewer gender minority clients opted to use this feature. Initial therapist matches on Talkspace are generated by an algorithm that incorporates user-reported preferences. Therapists can also self-identify areas of expertise that display on their profile, with 30% of the therapist network reporting experience working with lesbian, gay, bisexual, transgender, and queer communities [63]. The matching algorithm and therapists’ profile data may have reduced therapist changes for gender minority clients by helping them identify affirming providers. However, even with these safeguards, most gender minority clients did not improve at the same rate as men and women. Their depressive symptom trajectories may be influenced by a confluence of factors, including ongoing minority stress that hinders symptom improvement [45,64,65] and anticipated and past experiences of discrimination that can shape help-seeking behaviors, even when a better match might be available. Extant research shows that some gender minority clients compromise their care needs by remaining with providers who can offer either a specific clinical specialization or gender-affirming expertise, but not both [66]. In this study, it is possible that when working with their therapist, gender minority clients were satisfied with only one of these treatment domains, which could help explain the dichotomy of lower therapist-switching rates alongside slower symptom improvement. Indeed, finding the ideal provider with a combination of expertise, identity-affirmation, and service type has been described by some as “finding a unicorn” [67].

While the Talkspace matching algorithm considers some client preferences, it does not consider identity-based characteristics of therapists and restricts therapist gender preferences to “male” or “female.” These constraints may overlook factors that are particularly important to gender minority clients. The current study did not examine therapist qualities, including their formal training or self-reported experience working with gender minority clients, or client satisfaction data. However, some insight can be drawn from clients’ reported reasons for discontinuing treatment. Reasons reflected both positive outcomes and service-related barriers, including those known to disproportionately impact gender minority clients (eg, cost; service fit and effectiveness). Because gender minority clients generally report lower satisfaction with their care [68], more unmet expectations and unmet needs [20,69], and barriers to service acceptability [14], future research should work to incorporate provider ratings and areas of specialization to help clarify the experiences of gender minority clients who did not change therapists. Targeted outreach and clear messaging outlining the specific benefits of the therapist change feature for gender minority clients may encourage greater access. Improved engagement with this feature may lead to better therapeutic outcomes by ensuring that gender minority clients feel empowered to find a provider who meets both their clinical and identity-affirming needs.

### Strengths and Limitations

Strengths of the study included the naturalistic observation of treatment engagement and clinical outcomes on a large real-world telehealth platform. Gender was assessed with a single question, rather than the recommended 2-step measure that more accurately identifies gender minority individuals. Although single-item measures are common in general clinical settings because they reduce burden, they can misclassify people with transgender experience who do not identify as transgender. Furthermore, they fail to explicitly identify cisgender people who cannot be distinguished from transgender respondents selecting “male” or “female.” However, research shows that only a small proportion of transgender individuals avoid disclosing their identity on such measures [35]. Misclassification may only impact a small subset of Talkspace clients, but this limitation should still be considered when interpreting gender subgroup differences. Clients’ and therapists’ other sociodemographic factors may have influenced engagement. For example, there are known racial and ethnic disparities in access to and engagement with mental health care, including DMH services [70,71], but an analysis including these factors was not feasible due to large amounts of missing demographic data (eg, close to 86% of the sample did not provide information about their race or ethnicity). This study used the available data that may have contributed to treatment engagement and described reasons why clients canceled Talkspace services. Sensitivity analyses suggested that some of the treatment response observed in the full sample may be disproportionately driven by clients who discontinued treatment after showing initial steep symptom improvement, potentially inflating estimates of average treatment benefit. Missing data in later weeks may have been due to clients choosing not to fill out the surveys, leaving the platform after achieving adequate symptom improvement before week 15, discontinuing due to issues of fit or perceived effectiveness, or other reasons captured in cancellation data. To account for the missing data, our statistical approach used listwise deletion for all analyses except the multilevel modeling. The use of multilevel models accounted for unequal and smaller sample sizes of gender minorities.

### Future Directions

Future research may explore strategies to adapt digital interventions to better meet the needs of diverse gender identities, potentially incorporating specific modules on minority stress, identity affirmation, and navigating discrimination in healthcare settings. Treatment engagement and clinical outcomes of gender minority clients may be examined on other DMH platforms, purposeful sampling techniques may be used to increase sample sizes of gender minority clients, and inclusion of a control group would help to strengthen claims of efficacy. Future studies may also examine how sociodemographic factors impact treatment engagement and efficacy.

### Conclusions

Our study found that while clinical outcomes improved over time, some gender minority clients experienced slower symptom reduction compared to men and women. Depression severity decreased more slowly among transgender women, nonbinary, and gender-diverse clients. By week 15, PHQ-8 scores declined by an average of 8.7 points among men and women, compared to 5.7, 4.7, and 4.4 points among gender-diverse, nonbinary, and transgender women clients, respectively. On average, men and women clients of Talkspace also had higher rates of treatment response, symptom remission, and clinically significant symptom reduction than gender minority clients. We focused on gender minority clients to provide insight into their engagement with DMH services, the impacts of this engagement on clinical outcomes, and the need for more effective approaches to their care.

## Supplementary material

10.2196/75290 Multimedia Appendix 1 Multilevel linear models of depressive symptom trajectories among Talkspace clients (2017-2021) who completed the 15-week study period.

10.2196/75290 Multimedia Appendix 2 Adjusted 15-week depressive symptom trajectories by gender estimated among Talkspace clients (2017-2021) who completed the 15-week study period.

10.2196/75290 Multimedia Appendix 3 Multilevel linear models of depressive symptom trajectories among Talkspace clients (2017-2021) grouped by final 8-item Patient Health Questionnaire (PHQ-8) assessment week.

10.2196/75290 Multimedia Appendix 4 Adjusted 15-week depressive symptom trajectories by gender estimated among Talkspace clients (2017-2021) grouped by final 8-item Patient Health Questionnaire (PHQ-8) assessment week.

10.2196/75290 Multimedia Appendix 5 Reported reasons for service cancellation among Talkspace clients (2017-2021) who discontinued before week 15.

10.2196/75290 Checklist 1 STROBE checklist.

## Abbreviations

DMH: digital mental health
HIPAA: Health Insurance Portability and Accountability Act
PHQ-8: 8-item Patient Health Questionnaire
STROBE: Strengthening the Reporting of Observational Studies in Epidemiology
