# Supplementary material for: Gender Differences in Trajectories of Depressive Symptoms Among Talkspace Clients: Naturalistic Observational Study
Source: JMIR Form Res. 2025 Dec 3;9:e75290. doi: 10.2196/75290 (PMC12675994; doi:10.2196/75290)
Supplement: Multimedia Appendix 1 [file formative-v9-e75290-s001.docx]

| **Multimedia Appendix 1.** Multilevel linear models of depressive symptom trajectories among Talkspace clients (2017-2021) who completed the 15-week study period^a^ | | | |
| --- | --- | --- | --- |
|  | *b*^b^ | SE | *p* |
| **Intercept** | 3.10 |  |  |
| **Week number** | -1.01 | 0.05 | <0.001 |
| **Gender^c^** |  |  |  |
| Transgender men | 0.95 | 0.93 | 0.31 |
| Transgender women | -0.28 | 0.46 | 0.55 |
| Nonbinary | 0.28 | 0.68 | 0.68 |
| Gender diverse | 0.83 | 0.47 | 0.08 |
| Women | -0.12 | 0.12 | 0.32 |
| **Week number x Gender^c^** |  |  |  |
| Week number x Transgender men | 0.26 | 0.43 | 0.54 |
| Week number x Transgender women | 0.76 | 0.33 | 0.02 |
| Week number x Nonbinary | 0.03 | 0.29 | 0.91 |
| Week number x Gender diverse | 0.22 | 0.19 | 0.25 |
| Week number x Women | 0.09 | 0.05 | 0.08 |
| **Intake PHQ-8 score** | 0.69 | 0.01 | <0.001 |
| Notes: |  |  |  |
| 1. Model 2 re-estimated using only data from the subgroup of clients who submitted a PHQ-8 survey during the week 15 assessment period | | | |
| b. *b* represents the raw/unstandardized regression coefficient | | | |
| c. Reference group: men | | | |
