# Supplementary figures and images for: Gender Differences in Trajectories of Depressive Symptoms Among Talkspace Clients: Naturalistic Observational Study
Source: JMIR Form Res. 2025 Dec 3;9:e75290. doi: 10.2196/75290 (PMC12675994; doi:10.2196/75290)

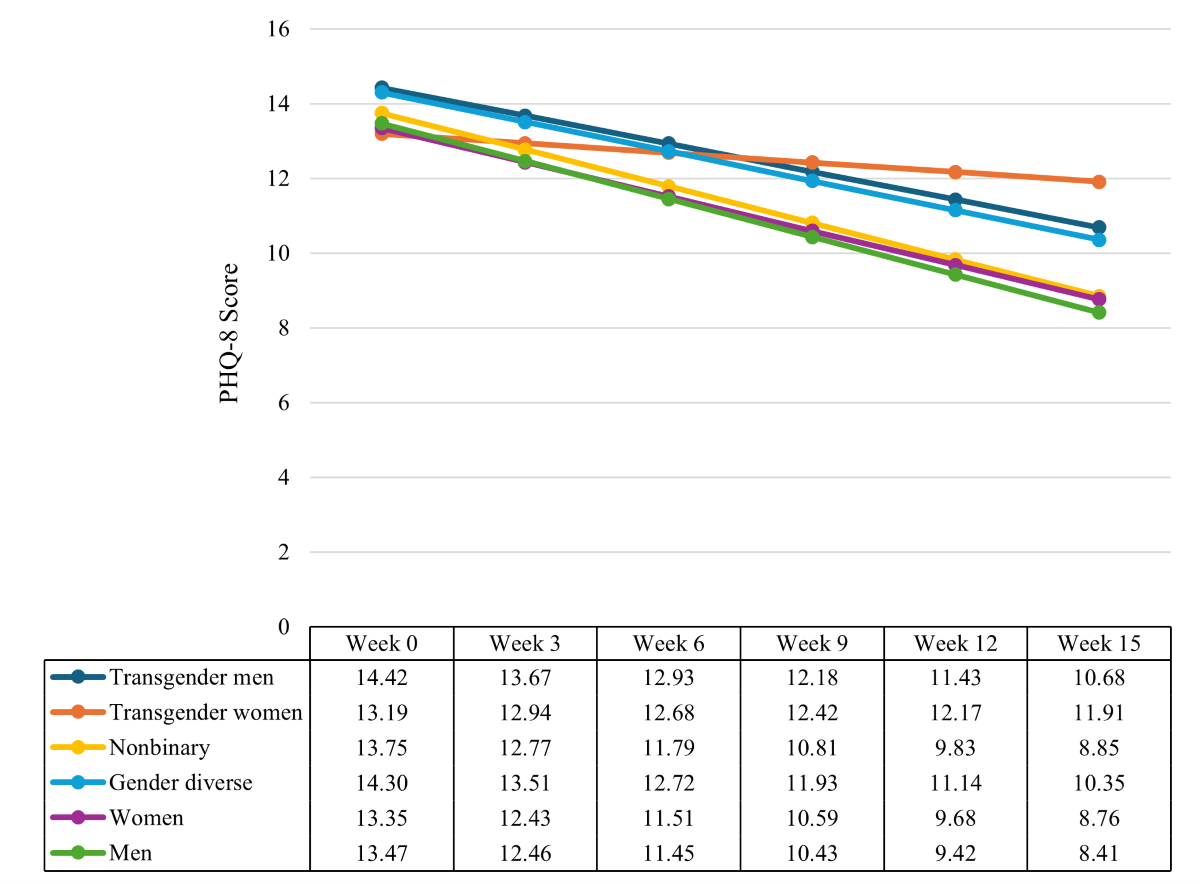

Supplement: Multimedia Appendix 2 [file formative-v9-e75290-s002.png]

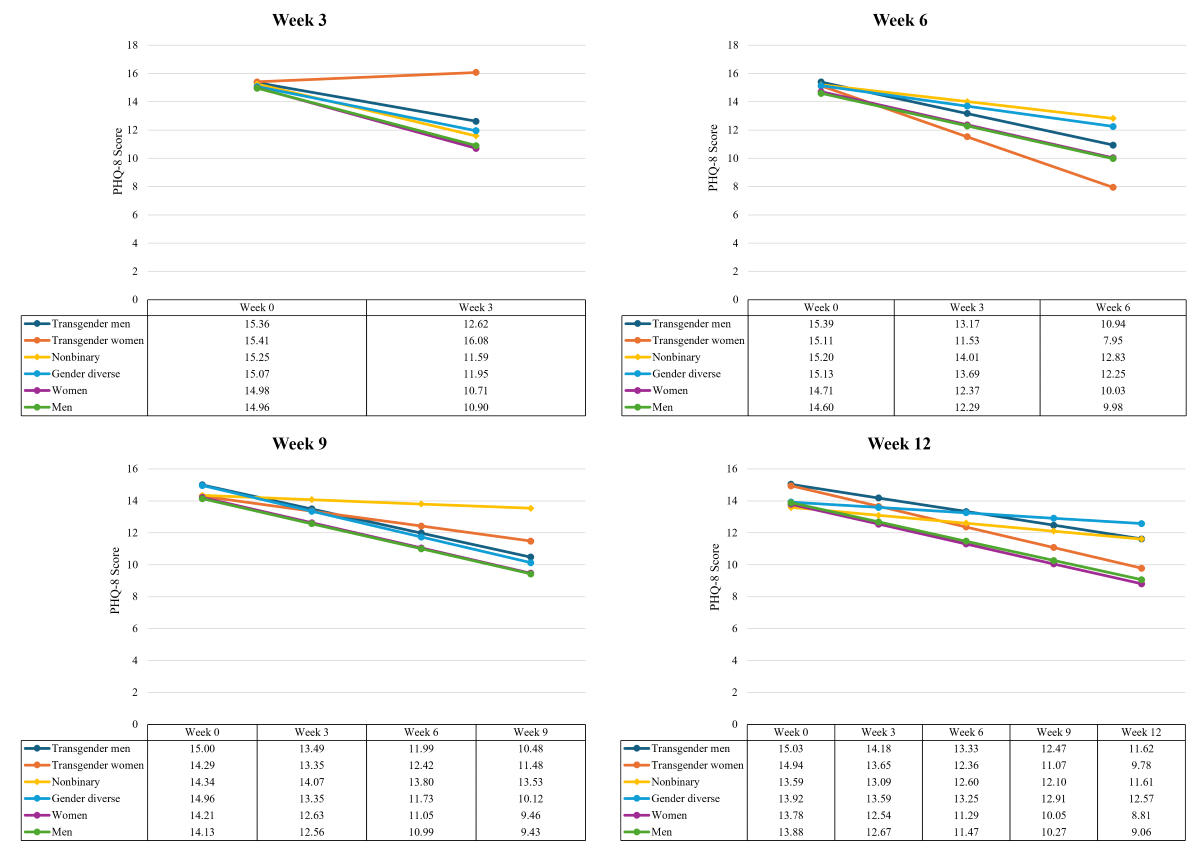

Supplement: Multimedia Appendix 4 [file formative-v9-e75290-s004.png]
