# Supplementary material for: Gender Differences in Trajectories of Depressive Symptoms Among Talkspace Clients: Naturalistic Observational Study
Source: JMIR Form Res. 2025 Dec 3;9:e75290. doi: 10.2196/75290 (PMC12675994; doi:10.2196/75290)
Supplement: Multimedia Appendix 3 [file formative-v9-e75290-s003.docx]

| **Multimedia Appendix 3**. Multilevel linear models of depressive symptom trajectories among Talkspace clients (2017-2021) grouped by final PHQ-8 assessment week^a^ | | | | | | | | | | | | | | | |
| --- | --- | --- | --- | --- | --- | --- | --- | --- | --- | --- | --- | --- | --- | --- | --- |
|  | Week 3 | | |  | Week 6 | | |  | Week 9 | | |  | Week 12 | | |
|  | *b*^b^ | SE | *p* |  | *b*^b^ | SE | *p* |  | *b*^b^ | SE | *p* |  | *b*^b^ | SE | *p* |
| **Intercept** | 1.25 |  |  |  | 2.25 |  |  |  | 2.23 |  |  |  | 3.11 |  |  |
| **Week number** | -4.06 | 0.13 | <0.001 |  | -2.31 | 0.10 | <0.001 |  | -1.57 | 0.08 | <0.001 |  | -1.20 | 0.07 | <0.001 |
| **Gender^c^** |  |  |  |  |  |  |  |  |  |  |  |  |  |  |  |
| Transgender men | 0.40 | 0.09 | <0.001 |  | 0.80 | 0.35 | 0.02 |  | 0.87 | 0.37 | 0.02 |  | 1.16 | 0.62 | 0.06 |
| Transgender women | 0.45 | 0.19 | 0.02 |  | 0.52 | 0.61 | 0.40 |  | 0.16 | 0.55 | 0.78 |  | 1.06 | 1.41 | 0.45 |
| Nonbinary | 0.29 | 0.09 | 0.001 |  | 0.60 | 0.27 | 0.03 |  | 0.21 | 0.66 | 0.75 |  | -0.29 | 0.87 | 0.74 |
| Gender diverse | 0.11 | 0.13 | 0.39 |  | 0.54 | 0.23 | 0.02 |  | 0.83 | 0.41 | 0.04 |  | 0.05 | 0.51 | 0.93 |
| Women | 0.02 | 0.05 | 0.69 |  | 0.11 | 0.09 | 0.21 |  | 0.08 | 0.12 | 0.52 |  | -0.10 | 0.14 | 0.50 |
| **Week number x Gender^c^** |  |  |  |  |  |  |  |  |  |  |  |  |  |  |  |
| Week number x Transgender men | 1.32 | 0.94 | 0.16 |  | 0.08 | 0.64 | 0.90 |  | 0.06 | 0.46 | 0.89 |  | 0.35 | 0.56 | 0.53 |
| Week number x Transgender women | 4.73 | 1.52 | 0.002 |  | -1.28 | 1.20 | 0.29 |  | 0.63 | 0.20 | 0.002 |  | -0.09 | 0.80 | 0.92 |
| Week number x Nonbinary | 0.39 | 0.98 | 0.69 |  | 1.12 | 0.52 | 0.03 |  | 1.30 | 0.42 | 0.002 |  | 0.71 | 0.36 | 0.05 |
| Week number x Gender diverse | 0.94 | 0.56 | 0.10 |  | 0.87 | 0.35 | 0.01 |  | -0.04 | 0.35 | 0.90 |  | 0.87 | 0.32 | 0.006 |
| Week number x Women | -0.21 | 0.15 | 0.15 |  | -0.03 | 0.11 | 0.79 |  | -0.01 | 0.09 | 0.87 |  | -0.04 | 0.08 | 0.60 |
| **Intake PHQ-8 score** | 0.90 | 0.01 | <0.001 |  | 0.81 | 0.01 | <0.001 |  | 0.79 | 0.01 | <0.001 |  | 0.71 | 0.02 | <0.001 |
| Notes: | | | | | | | | | | | |  |  |  |  |
| a. Model 2 re-estimated separately among clients grouped by final PHQ-8 assessment week (i.e., the last week a PHQ-8 score was available for each client, indicating the final survey they completed) | | | | | | | | | | | |  |  |  |  |
| b. *b* represents the raw/unstandardized regression coefficient | | | | | | | | | | | |  |  |  |  |
| c. Reference group: men | | | | | | | | | | | |  |  |  |  |
