# Supplementary material for: Gender Differences in Trajectories of Depressive Symptoms Among Talkspace Clients: Naturalistic Observational Study
Source: JMIR Form Res. 2025 Dec 3;9:e75290. doi: 10.2196/75290 (PMC12675994; doi:10.2196/75290)
Supplement: Multimedia Appendix 5 [file formative-v9-e75290-s005.docx]

| **Multimedia Appendix 5.** Reported reasons for service cancellation^a^ among Talkspace clients (2017-2021) who discontinued before week 15. | | | | | | | |
| --- | --- | --- | --- | --- | --- | --- | --- |
|  | Total Sample | Transgender men | Transgender women | Nonbinary | Gender diverse | Women | Men |
|  | n (%) | n (%) | n (%) | n (%) | n (%) | n (%) | n (%) |
|  | 3450 (100) | 9 (0.30) | 5 (0.10) | 13 (0.40) | 32 (0.93) | 2685 (77.83) | 706 (20.46) |
|  |  |  |  |  |  |  |  |
| Cancellation reason^b^ |  |  |  |  |  |  |  |
| Service Fit/Perceived Effectiveness | 677 (18.34) | 0 (0) | 3 (50.0) | 2 (15.38) | 13 (33.33) | 525 (18.35) | 134 (17.59) |
| Cost | 1431 (38.77) | 4 (40.0) | 0 (0) | 10 (76.92) | 15 (38.46) | 1121 (39.18) | 281 (36.88) |
| Prefer in-person care | 141 (3.82) | 1 (10.0) | 1 (16.67) | 0 (0) | 5 (12.82) | 103 (3.6) | 31 (4.07) |
| Not enough time/did not use enough | 49 (1.33) | 0 (0) | 1 (16.67) | 0 (0) | 1 (2.56) | 41 (1.43) | 6 (0.79) |
| Feel better/met goal | 928 (25.14) | 4 (40.0) | 0 (0) | 0 (0) | 5 (12.82) | 705 (24.64) | 214 (28.08) |
| Tech issues | 123 (3.33) | 0 (0) | 0 (0) | 1 (7.69) | 0 (0) | 90 (3.15) | 32 (4.2) |
| Other | 58 (1.57) | 0 (0) | 1 (16.67) | 0 (0) | 0 (0) | 46 (1.61) | 11 (1.44) |
| No reason provided | 284 (7.69) | 1 (10.0) | 0 (0) | 0 (0) | 0 (0) | 230 (8.04) | 53 (6.96) |
| 1. Cancellation data were obtained through a survey presented when participants selected to pause or cancel Talkspace services. Linked cancellation data were available for 6,287 of 20,156 (31.19%) participants. Reasons for service cancellation were examined among 3,450 (17.11%) participants who canceled within 60 days of their final survey and discontinued before week 15. Participants were excluded if they canceled: 1) after 60 days of their final survey (n=2,491; 12.36%), indicating cancellation timing outside of the study period, or; 2) within 60 days of their final survey, but completed the 15-week study period (n=346; 1.72%). Linked cancellation data were not available for 13,869 (68.81%) participants. | | | | | | | |
| 1. Cancellation reason totals may exceed the sample n as participants could provide more than one reason. | | | | | | | |
